# Trends, Demographic Characteristics and Seasonal Patterns of Rectal Prolapse Surgery in Japan: A Nationwide Claims‐Based Analysis From 2014 to 2023

**DOI:** 10.1002/ags3.70242

**Published:** 2026-06-26

**Authors:** Masamitsu Kido, Tomohiro Arita, Hiroki Shimizu, Jun Kiuchi, Kenji Nanishi, Katsutoshi Shoda, Hiroyuki Okimura, Atsuko Fujihara, Kengo Yoshii, Atsushi Shiozaki

**Affiliations:** ^1^ Department of Orthopedic Surgery Inage Hospital Chiba Japan; ^2^ Department of Surgery, Division of Digestive Surgery, Graduate School of Medical Science Kyoto Prefectural University of Medicine Kyoto Japan; ^3^ First Department of Surgery, Faculty of Medicine University of Yamanashi Yamanashi Japan; ^4^ Department of Obstetrics and Gynecology, Graduate School of Medical Science Kyoto Prefectural University of Medicine Kyoto Japan; ^5^ Department of Urology, Graduate School of Medical Science Kyoto Prefectural University of Medicine Kyoto Japan; ^6^ Department of Mathematics and Statistics in Medical Sciences Kyoto Prefectural University of Medicine Kyoto Japan

**Keywords:** Japan, laparoscopic surgery, national database, rectal prolapse, surgical epidemiology

## Abstract

**Aim:**

This study investigated nationwide trends, demographic characteristics, and seasonal patterns of rectal prolapse surgery in Japan, using healthcare claims databases.

**Methods:**

Surgical data were extracted from the National Database of Health Insurance Claims (NDB) from 2014 to 2023. Procedures were classified by surgical approach as conventional or laparoscopic. Crude and age‐adjusted rates per 100 000 person‐years were calculated. Temporal trends were assessed using linear and Poisson regression. Seasonal variations (April 2019–March 2024) were evaluated using one‐way analysis of variance with post hoc comparisons, and correlations with meteorological variables were examined using Pearson's correlation coefficients.

**Results:**

Over the decade, a total of 62 407 overall surgeries were performed, with an average annual rate of 4.7 per 100 000 person‐years and an overall female‐to‐male ratio of 6.9:1. Overall surgery rates peaked at ages 85–89 in males (5.5 per 100 000 person‐years) and females (59.1). While age‐adjusted overall and conventional surgeries declined significantly, laparoscopic surgeries significantly increased (all *p* < 0.01). Age‐adjusted overall surgery rates declined significantly in both sexes (RR = 0.960 for males, 0.983 for females, and 0.979 overall; each *p* < 0.0001), particularly among younger women aged 30–39 years (*p* < 0.0024). The mean seasonal overall surgeries exhibited distinct seasonal variation: 578.0 (SD 99.2) in spring, 440.0 (SD 85.9) in summer, 432.4 (SD 50.8) in autumn, and 544.4 (SD 71.8) in winter, and showed moderate negative correlations with temperature.

**Conclusion:**

This study provides contemporary epidemiological insights into surgical management of rectal prolapse in Japan.

## Graphical abstract

This study analyzed nationwide trends, demographic characteristics, and seasonal patterns of rectal prolapse surgery in Japan using healthcare claims data. From 2014 to 2023, age‐adjusted overall and conventional surgeries declined significantly, whereas laparoscopic surgeries increased significantly. Clear seasonal variation was observed, with significantly higher procedure volumes in spring and winter, and moderate inverse correlations with ambient temperature and humidity.

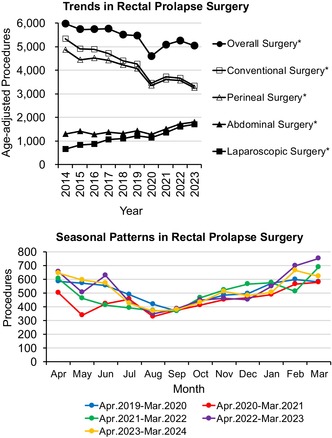

## 1 Introduction

Rectal prolapse is a debilitating pelvic floor disorder that significantly impairs a patient's quality of life due to symptoms such as fecal incontinence, constipation, and local discomfort [1, 2, 3]. The incidence rate in the general population is estimated to be 2.5 per 100 000 person‐years in the district of Central Finland [4]. Although the condition can occur in any demographic, it predominantly affects elderly women [1, 2, 3, 4, 5]. With the rapidly aging society in Japan, the number of rectal prolapse patients is expected to be increasing.

Surgery is the only curative treatment for this condition [1, 2, 3], and more than 100 surgical procedures have been reported to date. In recent years, laparoscopic ventral rectopexy (LVR) has gained worldwide popularity due to its lower recurrence rates and improved functional outcomes compared to perineal procedures [6, 7]. However, perineal approaches (e.g., Altemeier or Delorme procedures) are still widely valued for their minimal invasiveness, making them a suitable option for frail, high‐risk elderly patients. While clinical guidelines exist [1, 2, 3], it remains unclear how these surgical approaches are selected and applied across different age groups in real‐world clinical practice on a nationwide scale.

Furthermore, anecdotal evidence, and limited studies suggest that the incidence of rectal prolapse may fluctuate with the seasons, potentially influenced by environmental factors such as temperature‐related respiratory or bowel habits. However, comprehensive epidemiological data correlating surgical volume with meteorological factors are lacking.

This study aimed to clarify the recent trends in rectal prolapse surgery in Japan using the National Database of Health Insurance Claims (NDB) [8], which captures over 95% of all insured medical care claims nationwide. Building on previous nationwide studies in gastroenterological and thoracic surgery [9, 10, 11, 12, 13], we specifically analyzed age‐related patterns in the selection of laparoscopic versus conventional approaches and identified seasonal variations in surgical incidence over the past decade.

## 2 Methods

### 2.1 Study Design and Data Sources

This retrospective observational epidemiological study utilized publicly available, anonymized databases. According to Japanese ethical guidelines for studies using de‐identified public data, Institutional Review Board approval and informed consent were not required. The study was based on the NDB Open Data Japan [14], which provides pre‐aggregated nationwide healthcare claims data released by the Ministry of Health, Labour and Welfare. To protect patient anonymity, procedure counts < 10 within any stratified category (e.g., by age, sex, or month) are systematically masked prior to public release, and the exact number of suppressed procedures is not disclosed. To ensure transparency, we calculated completeness rates, defined as the proportion of observable stratified data relative to the corresponding crude totals. This metric serves as a surrogate indicator of data availability and allows assessment of the potential impact of data masking on the analysis.

We extracted annual data on the number of rectal prolapse surgeries, stratified by sex and age using the NDB Open Data Japan (2014–2023) [14], an open‐access subset of the NDB. Population denominators for rate calculations were derived from Ministry of Internal Affairs and Communications statistics [15].

### 2.2 Selection Criteria for Rectal Prolapse Surgery and Classification

Rectal prolapse surgeries were classified according to surgical approach based on Japanese procedure codes. The following procedure codes were included:

K742 Rectal prolapse surgery (conventional)

1. Transperineal without bowel resection (e.g., Gant‐Miwa and Thiersch procedures).
2. Transperineal with bowel resection by Altemeier or Delorme procedures.
3. Abdominal with rectal fixation (rectopexy).
4. Abdominal with pelvic floor reconstruction.
5. Abdominoperineal (including bowel resection).

K742‐2 Rectal prolapse surgery (laparoscopic).

Therefore, we categorized rectal prolapse surgeries as follows:

- Conventional surgery: K742 (1) (2) (3) (4) (5).
- Laparoscopic surgery: K742‐2.
- Perineal surgery: K742 (1) (2).
- Abdominal surgery: K742 (3) (4) (5) and K742‐2.
- Overall surgery: K742 (1) (2) (3) (4) (5) and K742‐2.

In Japan's insurance reimbursement system, the primary coding distinction is between conventional (K742) and laparoscopic (K742‐2) procedures. Accordingly, the “conventional surgery” category was defined to align with this coding structure and to enable a consistent laparoscopic‐versus‐conventional comparison across the full study period. Subgroup analyses of perineal and abdominal surgeries were conducted where data permitted; however, due to substantial data masking and low completeness in conventional abdominal subcategories [K742 (3–5)], these results are presented descriptively rather than as primary analytical outcomes.

Because of changes in coding definitions, perineal procedures were distinguishable by the presence or absence of bowel resection (K742 (1) and (2)) only from 2016 onward.

### 2.3 Descriptive Analysis

We calculated the annual average numbers and rates of overall rectal prolapse surgery per 100 000 population. We also computed the overall male‐to‐female ratio of procedure rates. Demographic peak analysis was conducted by assessing sex‐ and age‐stratified (in 5‐year increments) procedure rates per 100 000 person‐years.

### 2.4 Temporal Trend Analysis

Annual trends for procedure counts and rates were evaluated from 2014 to 2023. Procedure counts were analyzed using linear regression models. For procedure rates, Poisson regression models were fitted with the fiscal year entered as a continuous explanatory variable. Relative risks (RRs) were estimated for males, females, and the combined population, both overall and within age‐stratified subgroups. An RR of < 1.0 indicates a year‐on‐year decrease in the procedure rate per 100 000 person‐years, an RR > 1.0 indicates an increase, and an RR = 1.0 represents no annual change.

### 2.5 Seasonal Analysis

Seasonal patterns were examined using monthly data on overall procedure counts from April 2019 to March 2024, following previously reported methods [13]. Monthly counts were standardized to a 30‐day basis. Seasons were defined according to the Japan Meteorological Agency classification: spring (March–May), summer (June–August), autumn (September–November), and winter (December–February) [16]. As aggregated nationwide meteorological data were unavailable, monthly meteorological parameters (temperature, barometric pressure, humidity, sunshine hours, and precipitation) were obtained from five major cities (Tokyo, Osaka, Nagoya, Fukuoka, and Sapporo) to serve as a proxy for national conditions [17].

### 2.6 Statistical Analyses

The number was adjusted for age by the direct method, based on the 2015 Japanese standard population [12]. Linear regression analysis was performed assessing annual changes in the age‐adjusted procedure counts. Poisson regression models were constructed to examine annual changes in the age‐adjusted procedure rate per 100 000 population. The procedure number was set as the objective variable, and the observation time‐point (year) and sex were set as the explanatory variables. In addition, the population at each time‐point was considered by adding the person‐year population to the model as an offset. Moreover, annual trend analysis of the subgroups was performed using age‐ and sex‐stratified samples.

A seasonal analysis was performed using a one‐way analysis of variance (ANOVA), followed by post hoc comparisons using the Tukey–Kramer HSD test across the four seasons. Correlations between overall rectal prolapse surgeries and meteorological variables among 60 monthly time points from April 2019 to March 2024 were evaluated using Pearson's correlation coefficient. The correlation between the national monthly rectal prolapse surgery counts and meteorological data from the five major cities was analyzed because of the absence of city‐specific incidence or national meteorological data.

All statistical analyses were performed using R version 4.5.2 Statistical Computing Program (R Foundation; www.r‐project.org) and EZR (Jichi Medical University Saitama Medical Center, Saitama, Japan) software programs [18]. Statistical significance was set at two‐sided *p*‐values of < 0.05 for the linear regression models. For Poisson regression models, significance thresholds were adjusted for multiple comparisons using the Bonferroni correction: *p* < 0.0167 (0.05/3) for all ages analyses; *p* < 0.0024 (0.05/21) for age‐stratified analyses of overall and conventional surgery; and *p* < 0.0028 (0.05/18) for age‐stratified analyses of laparoscopic surgery.

## 3 Results

### 3.1 Overview

**FIGURE 1:**
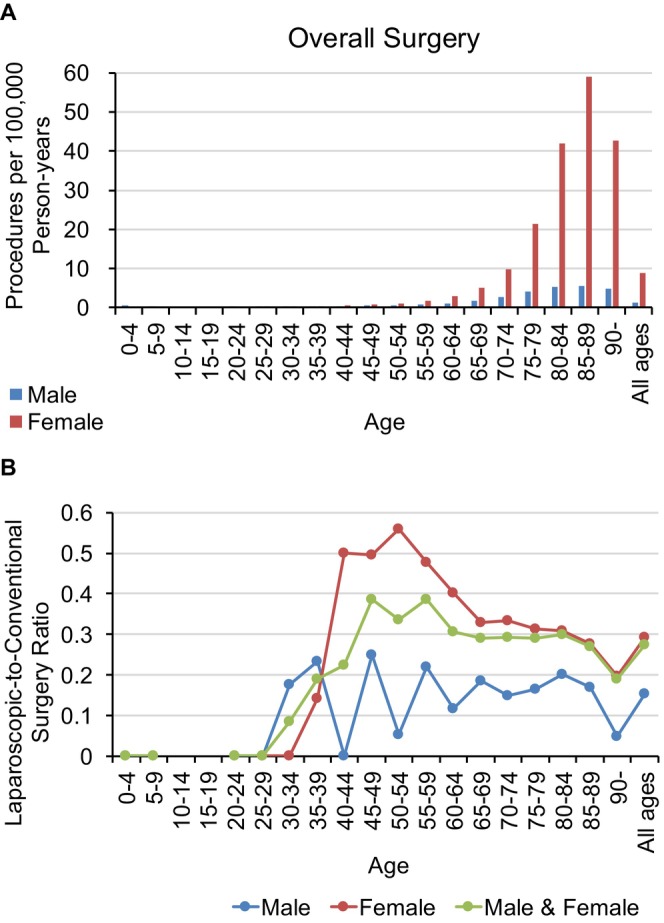
(A) Age‐stratified distribution of rectal prolapse surgeries among males and females, expressed per 100 000 person‐years from 2014 to 2023. (B) Age‐stratified distribution of laparoscopic‐to‐conventional surgery ratios among males, females, and both sexes.

Between 2014 and 2023, a total of 62 407 rectal prolapse surgeries were performed across Japan, equating to a crude rate of 4.7 per 100 000 person‐years. The overall female‐to‐male ratio was 6.9:1. Age‐stratified analysis of 59 109 procedures (completeness rate: 94.7%) revealed a unimodal peak distribution in overall surgery rates, peaking in the 85–89 age group (Figure 1A). In the 85–89 age group, the annual procedure rates per 100 000 person‐years were 5.5 for males, 59.1 for females, and 45.4 for both sexes combined. Overall, 91.6% of all surgeries occurred in patients aged ≥ 65 years.

Specifically, age‐stratified analysis revealed a unimodal peak distribution in conventional surgery rates, peaking in the 85–89 age group (Figure S1A). In the 85–89 age group, the annual procedure rates per 100 000 person‐years were 4.7 for males, 46.3 for females, and 35.7 for both sexes combined. As for laparoscopic surgery rates, a unimodal peak distribution was also observed, peaking in the 85–89 age group (Figure S1B). In the 85–89 age group, the annual procedure rates per 100 000 person‐years were 0.8 for males, 12.8 for females, and 9.7 for both sexes combined. The age‐stratified distribution of perineal and abdominal procedures is presented in Figure S1C,D, respectively.

The laparoscopic‐to‐conventional surgery ratio across different age groups are presented in Figure 1B. The overall ratio for all ages and sexes was 0.275. A breakdown by gender revealed a higher overall ratio in females (0.293) compared to males (0.154).

A marked increase was observed beginning in the 30–34 age group. In the female cohort, the ratio rose sharply in the fourth decade of life, reaching a peak of 0.560 in the 50–54 age group. Following this peak, the ratio gradually declined with advancing age, falling to 0.196 in the ≥ 90 age group. In contrast, the male cohort exhibited a more fluctuating pattern with generally lower ratios. From age 60 onwards, the male ratio hovered between 0.11 and 0.22, eventually decreasing to 0.048 in the ≥ 90 age group.

### 3.2 Procedural Breakdown in 2023

In fiscal year 2023, a total of 6401 rectal prolapse surgeries were performed in Japan. Age‐ and sex‐stratified counts were available for 6041 procedures (completeness rate: 94.4%), which are presented in Table 1.

**TABLE 1:** Surgical procedures for rectal prolapse surgery in Japan, 2023.

Procedure	Crude count (%)	Sex	Age‐ and sex‐stratified surgery count										Completeness rate (%)
Conventional			< 10	10s	20s	30s	40s	50s	60s	70s	80s	≥ 90	
Perineal surgery without bowel resection	3078 (48.1%)	M	11	—	—	—	14	37	45	109	109	27	95.1
		F	—	—	—	—	25	52	109	539	1347	504	
Perineal surgery with bowel resection	1060 (16.6%)	M	—	—	—	—	—	—	10	44	26	—	93.7
		F	—	—	—	—	—	10	43	207	488	165	
Abdominal surgery with rectal fixation (rectopexy)	127 (2.0%)	M	—	—	—	—	—	—	—	—	—	—	85.8
		F	—	—	—	—	—	—	—	29	61	19	
Abdominal surgery with pelvic floor reconstruction	35 (0.5%)	M	—	—	—	—	—	—	—	—	—	—	0.0
		F	—	—	—	—	—	—	—	—	—	—	
Abdominoperineal surgery including bowel resection	35 (0.5%)	M	—	—	—	—	—	—	—	—	—	—	28.6
		F	—	—	—	—	—	—	—	—	10	—	
Laparoscopic													
Laparoscopic surgery	2066 (32.3%)	M	—	—	—	11	12	13	26	48	38	12	96.9
		F	—	—	—	—	29	57	130	463	923	239	
Overall surgery	6401 (100.0%)	M	11	—	—	11	26	50	81	201	173	39	94.4
		F	—	—	—	—	54	119	282	1238	2829	927	

*Note:* (1) To protect patient anonymity, the NDB Open Data Japan [14] omits procedure counts of fewer than 10 per reporting unit; such cells are indicated by “‐”. (2) Completeness rate = (sum of reported cells ≥ 10) ÷ crude count, reflecting the proportion of observable data after suppression.

Abbreviations: F, female; M, male.

The procedural distribution included: 3078 conventional perineal surgery without bowel resection (48.1%), 2066 laparoscopic surgery (32.3%), 1060 conventional perineal surgery with bowel resection (16.6%), 127 conventional abdominal surgery with rectal fixation (rectopexy) (2.0%), 35 conventional abdominal surgery with pelvic floor reconstruction (0.5%), and 35 conventional abdominoperineal surgery including bowel resection (0.5%).

Notably, conventional surgery accounted for 66.3% of overall surgeries (4335/6401), while laparoscopic surgery accounted for 32.3% (2066/6401).

### 3.3 Annual Trend Analysis From 2014 to 2023

#### 3.3.1 Annual Trends in Procedure Counts

**FIGURE 2:**
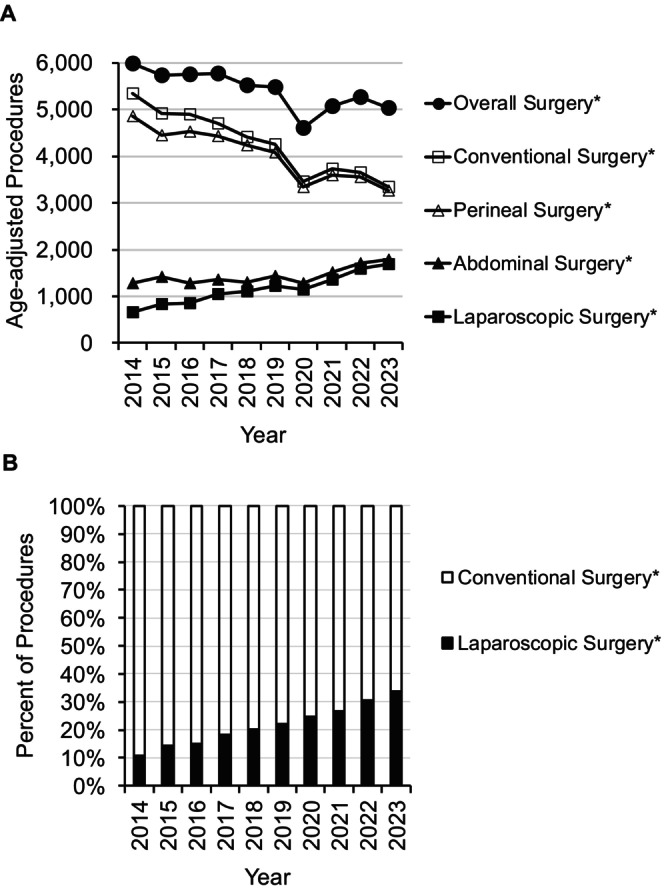
Trends in age‐adjusted rectal prolapse surgeries (2014–2023) in Japan. Surgical procedures are further categorized into conventional/laparoscopic and perineal/abdominal approaches. * Indicates a statistically significant trend (*p* < 0.05).

Over the study period, a significant decreasing trend was observed in the age‐adjusted overall rectal prolapse surgeries (*p* = 0.0044), including conventional and perineal surgery (both *p* < 0.0001), while laparoscopic and abdominal surgeries showed increasing trends (both *p* < 0.0001) (Figure 2A). Notably, the proportion of laparoscopic surgeries among overall surgeries increased annually (Figure 2B).

#### 3.3.2 Annual Trends in Procedure Rates

**FIGURE 3:**
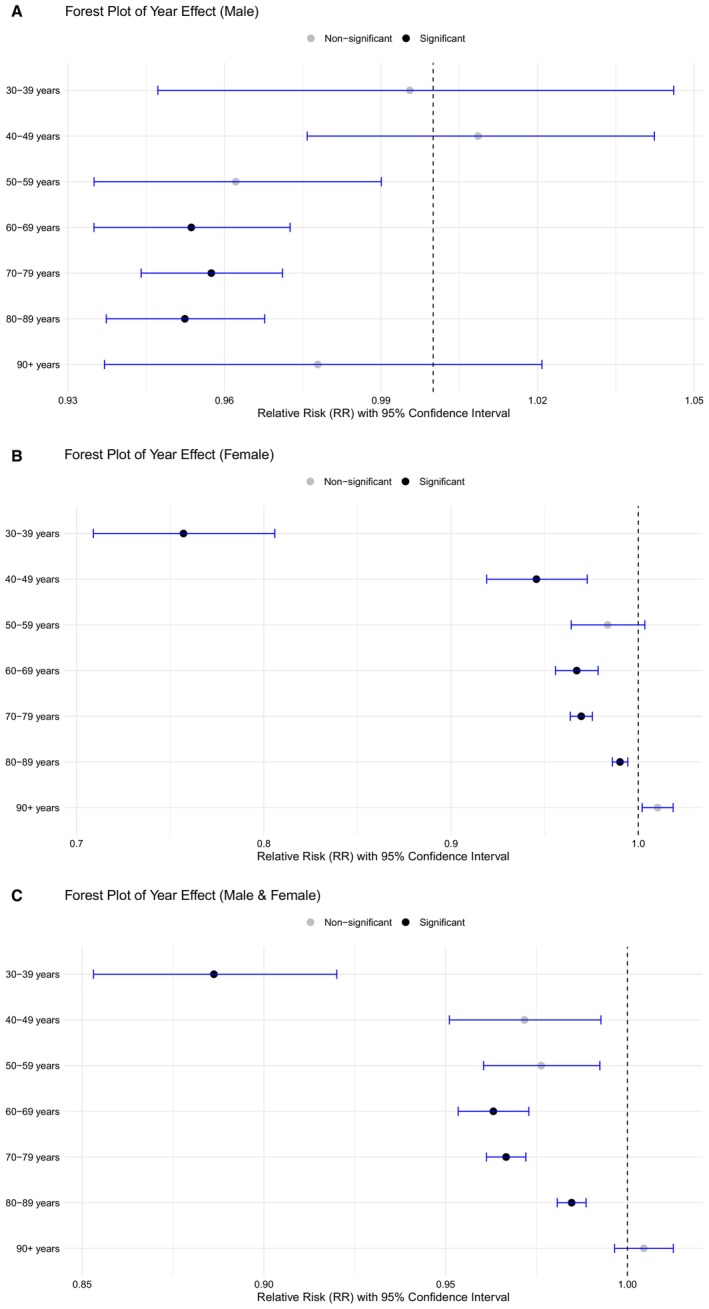
Relative risks (RRs) for age‐stratified rectal prolapse surgeries per 100 000 person‐years, derived from Poisson regression models: Males (A), females (B) and both sexes combined (C). Black dots represent significant, and gray dots represent non‐significant. A dashed line at RR = 1.0 represents no annual change in procedure rate. CI, confidence interval; RR, relative risk.

Age‐adjusted overall surgery rates across all age groups per 100 000 person‐years demonstrated statistically significant annual decreases among males, females and both sexes (RR = 0.960, 0.983, and 0.979, respectively; each *p* < 0.0001) (Table 2). Subgroup analyses further revealed significant decreases among males aged 60–89 years (RR range: 0.952 to 0.957; all *p* < 0.0001), females aged 30–49 and 60–89 years (RR range: 0.757 to 0.990; all *p* < 0.001), and both sexes aged 30–39 and 60–89 years (RR range: 0.886 to 0.985; all *p* < 0.0001) (Figure 3 and Table S1).

**TABLE 2:** Poisson regression analysis of annual trends in age‐adjusted rectal prolapse surgery rates across all ages per 100 000 person‐years.

Outcome	Sex	RR	95% CI (low)	95% CI (high)	*p*
Overall surgery	Male	0.960	0.953	0.968	< 0.0001
	Female	0.983	0.979	0.986	< 0.0001
	Male and female	0.979	0.976	0.982	< 0.0001
Conventional surgery	Male	0.937	0.929	0.945	< 0.0001
	Female	0.951	0.948	0.955	< 0.0001
	Male and female	0.949	0.946	0.952	< 0.0001
Laparoscopic surgery	Male	1.109	1.085	1.134	< 0.0001
	Female	1.098	1.090	1.106	< 0.0001
	Male and female	1.098	1.091	1.105	< 0.0001

*Note:* RR reflects the relative risk per one‐year increase (time variable entered as continuous).

Abbreviations: CI, confidence interval; RR, relative risk.

Conventional and laparoscopic surgery rates across all age groups per 100 000 person‐years showed significant decreasing and increasing trends, respectively (Table 2). Notably, subgroup analyses revealed significant decreases in conventional surgeries among younger individuals aged 30–39 years for both sexes (RR: 0.840; *p* < 0.0001), while significant increases in laparoscopic surgeries among younger males aged 40–49 years (RR: 1.609; *p* < 0.0001) and older females aged ≥ 90 years (RR: 1.162; *p* < 0.0001) (Figures S2 and S3; Tables S2 and S3).

### 3.4 Seasonal Analysis

**FIGURE 4:**
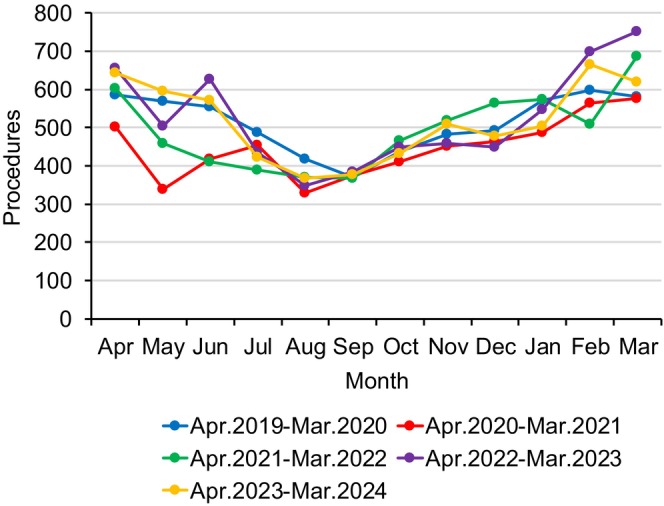
Monthly national rectal prolapse surgeries in 12 months from April 2019 to March 2024.

Monthly overall rectal prolapse surgeries (completeness rate: 97.8% [30 329/30 998]) showed clear seasonal variability (Figure 4). One‐way ANOVA confirmed a significant overall seasonal effect (*p* < 0.0001), with post hoc Tukey–Kramer HSD testing identified significant differences specifically between the spring and autumn, spring and summer, winter and summer, and winter and autumn seasons (all *p* < 0.01). The mean seasonal procedures were 578.0 (SD 99.2) in spring, 440.0 (SD 85.9) in summer, 432.4 (SD 50.8) in autumn, and 544.4 (SD 71.8) in winter.

**FIGURE 5:**
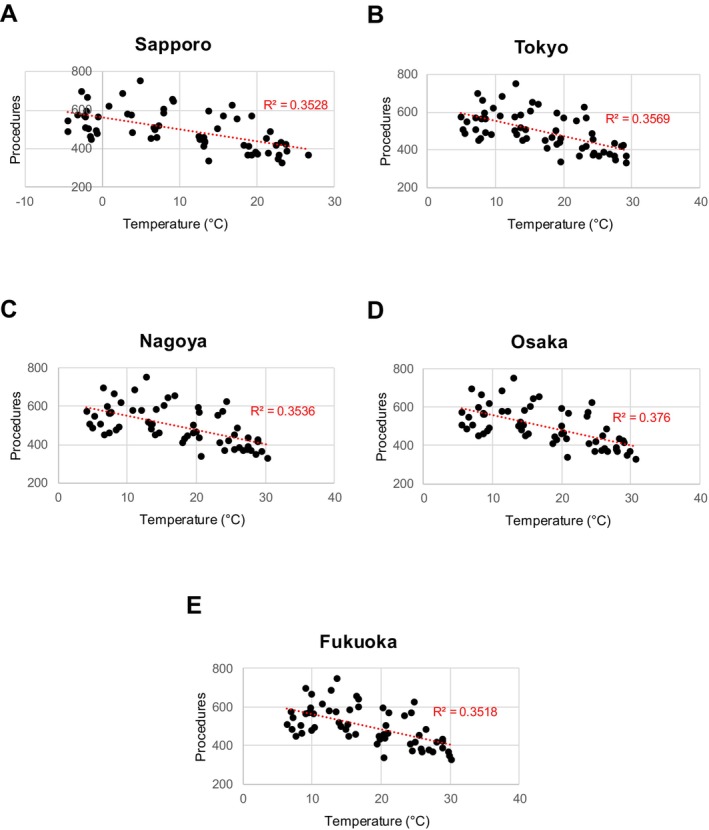
Scatter plots showing correlations between monthly mean temperature data from five geographically distributed major cities (A: Sapporo, B: Tokyo, C: Nagoya, D: Osaka, E: Fukuoka) and the corresponding nationwide monthly rectal prolapse surgery counts (*y*‐axis; standardized to a 30‐day basis) across 60 monthly time points from April 2019 to March 2024. Each data point represents 1 month. Because aggregated nationwide meteorological data are unavailable, city‐specific data were used as regional proxies for national conditions.

Correlation analyses across the five major cities revealed consistent moderate negative correlations between monthly surgery counts and both temperature and humidity, whereas mild‐to‐moderate positive correlations were noted with barometric pressure (Table 3). Specifically, Pearson correlation coefficients ranged from *r* = −0.59 to −0.61 for temperature and *r* = −0.40 to −0.57 for humidity across all five cities. The scatter plots illustrating the correlation between nationwide monthly surgery counts and city‐specific mean temperature are presented in Figure 5, where the y‐axis represents surgery counts standardized to a 30‐day basis and each data point corresponds to a single monthly observation (*n* = 60 time points). Because aggregated nationwide meteorological data are unavailable in Japan, city‐specific data from five geographically dispersed cities—spanning from Sapporo in the north (latitude 43° N) to Fukuoka in the south (latitude 33° N)—were used as regional proxies for national conditions. The consistency of correlation patterns across this climatically diverse range of cities supports the generalizability of these findings.

**TABLE 3:** Correlation between monthly national rectal prolapse surgeries and meteorological data across the five major cities in Japan.

	Temperature (°C)	Barometric pressure (hPa)	Humidity (%)	Daylight hours (h)	Precipitation (mm)	Location
Sapporo	−0.59	0.27	−0.43	−0.11	−0.14	43°3′ 43.49″ N, 141°21′15.8″ E
Tokyo	−0.60	0.39	−0.55	0.25	−0.23	35°40′52″ N, 139°46′0″ E
Nagoya	−0.59	0.45	−0.57	0.16	−0.23	35°10′ 53.04″ N, 136° 54′23.04″ E
Osaka	−0.61	0.46	−0.50	−0.09	−0.19	34°41′ 37.5″ N, 135° 30′7.6″ E
Fukuoka	−0.59	0.44	−0.40	−0.11	−0.26	33°35′24″ N, 130° 24′6.12″ E

*Note:* Available 60 monthly data were assessed from April 2019 to March 2024.

## 4 Discussion

This nationwide claims‐based study provides a comprehensive overview of the epidemiology of rectal prolapse surgery in Japan over the most recent decade (2014–2023), using a population‐level database that covers nearly all insured medical care. Several important findings emerged. First, rectal prolapse surgery was overwhelmingly concentrated among older adults, particularly elderly women, with a clear unimodal age distribution peaking in the late 80s. Second, age‐adjusted overall and conventional surgery rates declined significantly over time, whereas laparoscopic surgery increased steadily across almost all demographic subgroups. Third, rectal prolapse surgery demonstrated marked seasonal variation, characterized by significantly higher procedure volumes in spring and winter and moderate negative correlations with temperature and humidity.

Consistent with previous reports [1, 2, 3, 4, 5], our data confirmed that rectal prolapse surgery in Japan predominantly affects older adults; more than 90% of procedures were performed in patients aged ≥ 65 years, with a pronounced female predominance (female‐to‐male ratio of 6.9:1) (Figure 1A). The peak incidence in the 85–89 age group observed in both sexes underscores the strong association between rectal prolapse and age‐related factors, such as pelvic floor weakness, multiparity, and connective tissue degeneration. Notably, the peak age for females was slightly younger than that for males, which may reflect cumulative obstetric and hormonal influences unique to women.

As previously reported in England [5], Japan's rapidly aging population was expected to increase the absolute and relative burden of rectal prolapse surgery. However, our age‐adjusted trend analyses revealed a paradoxical decline in overall surgical rates, particularly for conventional procedures (Figure 2A). This finding suggests that demographic aging alone does not explain recent surgical trends but that multiple converging factors may be contributing. In addition to the transient decline observed in 2020, likely attributable to the COVID‐19 pandemic, several longer‐term mechanisms warrant consideration. First, the increasing adoption of laparoscopic surgery—reported to be associated with lower recurrence rates than perineal procedures [1, 2, 3]—may have reduced the need for repeat surgery, thereby contributing to the overall decline in surgical volume over time. Second, improvements in conservative management strategies for pelvic floor dysfunction, including pelvic floor muscle training, biofeedback therapy, and lifestyle modification, may have deferred or averted surgery in some patients. Third, increased awareness and earlier diagnosis of rectal prolapse at less advanced stages may have led to more conservative decision‐making in select patients. Particularly among women, this decline may also reflect recent changes in perinatal care, including improved pelvic floor protection and preventive strategies for pelvic floor disorders [19]. Furthermore, Japan's declining birth rate [20] may have contributed to a reduction in cumulative obstetric stress on the pelvic floor. In addition, increasing cesarean section rates aimed at improving maternal and fetal safety [21], consistent with global trends [22], may have further reduced obstetric trauma and thereby lowered the long‐term risk of rectal prolapse in this population. Planned cesarean deliveries on maternal request have also accounted for approximately 2.5% of births in the United States [23], and their effect in Japan will be considered in the future.

In contrast to the decline in conventional surgery, laparoscopic rectal prolapse surgery increased significantly over the study period. By 2023, laparoscopic procedures accounted for approximately one‐third of all rectal prolapse surgeries nationwide (Figure 2B and Table 1). This transition mirrors global trends toward minimally invasive surgery [5, 24, 25, 26] and reflects growing surgeon expertise, advances in laparoscopic instrumentation, and increasing acceptance of laparoscopic rectopexy even in older patients. However, our age‐stratified analysis revealed that patient selection remains highly dependent on age and gender.

While the overall proportion of laparoscopic surgery increased over the study period (Figure 2B), the laparoscopic‐to‐conventional surgery ratio in females exhibited a distinctive “inverted U‐shape” pattern (Figure 1B), peaking at 0.560 in the 50–54 age group and subsequently declining with advancing age. Within the Japanese insurance reimbursement system, the classification of “conventional surgery” includes not only abdominal rectopexy via laparotomy but also perineal procedures (e.g., Delorme or Altemeier). Importantly, approximately 95% of conventional surgeries recorded in 2023 were perineal procedures (4138 of 4335 procedures) (Table 1). Therefore, the observed decline in the laparoscopic‐to‐conventional ratio among older females likely reflects age‐specific surgical decision‐making. Specifically, laparoscopic ventral rectopexy (LVR), known for its lower recurrence rates and superior functional outcomes, is preferentially selected for middle‐aged patients to maintain quality of life and physical activity. In contrast, among super‐elderly patients (e.g., those ≥ 90 years, in whom the ratio decreased to 0.196), perineal approaches are more commonly favored because they impose less anesthetic and hemodynamic stress, despite their higher risk of recurrence.

Conversely, the laparoscopic‐to‐conventional surgery ratio in males was consistently lower than that in females (0.154 vs. 0.293, overall) (Figure 1B). This discrepancy may reflect sex differences in the pathophysiology of rectal prolapse. Whereas obstetric factors play a major role in females, prolapse in males is more often linked to long‐standing colorectal dysfunction or chronic pelvic floor weakness, and its underlying mechanisms remain incompletely understood [1, 27]. Consequently, surgical management in males may more frequently favor perineal approaches or be influenced by a higher burden of comorbidities at diagnosis.

Another novel finding of this study is the clear seasonal pattern in rectal prolapse surgery volume. Surgical counts were significantly lower in summer and autumn and higher in spring and winter, with moderate negative correlations between procedure volume and ambient temperature and humidity. Although rectal prolapse is not an acute condition, these findings suggest that symptom severity or healthcare‐seeking behavior is influenced by environmental factors. Several mechanisms may be hypothesized. Colder temperatures and lower humidity may affect bowel habits or increase symptom awareness, while seasonal factors may also influence patients' willingness to seek elective surgical care, particularly among older adults. In support of this hypothesis, our previous nationwide study demonstrated that digital evacuation procedures for severe constipation also showed a winter predominance with significant negative correlations with ambient temperature [13]. Although digital evacuation does not directly cause or detect rectal prolapse, the parallel seasonal pattern suggests that cold weather may exacerbate bowel symptoms—increasing straining and pelvic floor stress in susceptible individuals—which may in turn heighten symptom awareness and prompt surgical consultation during the colder months. An alternative explanation is that, as an elective procedure, rectal prolapse surgery may be preferentially scheduled during periods of lower operating room demand, such as when volumes of urgent procedures (e.g., cancer surgery) are reduced; whether competing demand for surgical resources contributes to the observed seasonal pattern warrants investigation in future studies. Together, these findings underscore the importance of seasonally tailored perioperative management and lifestyle guidance—such as maintaining adequate warmth and optimizing bowel habits—for patients with rectal prolapse, particularly during the winter months.

From a clinical perspective, the observed shift from conventional to laparoscopic surgery highlights the importance of maintaining and expanding minimally invasive surgical training, particularly in the context of an aging surgical workforce. From a health policy standpoint, the declining overall surgery rates despite population aging suggest that healthcare demand for rectal prolapse surgery may remain stable or even decrease, although absolute case numbers will likely remain substantial due to demographic pressures. Furthermore, recognition of seasonal variation may aid hospital resource planning, surgical scheduling, and workforce allocation, particularly in regions with a high proportion of elderly residents.

This study has several limitations. First, the NDB lacks detailed clinical information, such as prolapse severity, symptom duration, functional outcomes, or recurrence rates, precluding assessment of disease severity or surgical effectiveness. Second, laparoscopic procedures could not be further subclassified by operative technique (e.g., ventral mesh rectopexy versus posterior mesh rectopexy), as the NDB procedure code K742‐2 encompasses all laparoscopic approaches without further distinction. Third, the exclusion of procedures with fewer than 10 annual instances for privacy protection may have resulted in underestimation or overestimation of infrequently performed surgeries. This limitation has a negligible impact on the primary findings; however, it should be noted when interpreting results for low‐incidence subgroups. Fourth, seasonal correlation analyses were limited by the unavailability of aggregated nationwide meteorological data, necessitating the use of city‐specific data as regional proxies. Additionally, the findings should be interpreted as ecological correlations rather than individual‐level associations, and causal relationships cannot be inferred. Finally, non‐surgical management and untreated cases were not captured; therefore, our findings reflect surgical epidemiology rather than the true incidence of rectal prolapse.

In conclusion, this nationwide study demonstrates that rectal prolapse surgery in Japan is increasingly concentrated among elderly women, with a significant long‐term decline in age‐adjusted overall and conventional surgery rates despite population aging. Concurrently, laparoscopic surgery has expanded steadily and now represents a substantial proportion of surgical management. The presence of clear seasonal variation further underscores the complex interplay between environmental factors and surgical care utilization. These findings provide valuable epidemiological evidence to inform future surgical practice, training strategies, and healthcare policy in super‐aging societies.

## Author Contributions

**Hiroki Shimizu:** writing – review and editing. **Atsushi Shiozaki:** supervision. **Hiroyuki Okimura:** writing – review and editing. **Jun Kiuchi:** writing – review and editing. **Kenji Nanishi:** writing – review and editing. **Atsuko Fujihara:** writing – review and editing. **Katsutoshi Shoda:** methodology, writing – review and editing. **Kengo Yoshii:** writing – review and editing, validation. **Masamitsu Kido:** conceptualization, methodology, data curation, investigation, formal analysis, visualization, writing – original draft, writing – review and editing. **Tomohiro Arita:** conceptualization, formal analysis, writing – review and editing.

## Funding

The authors did not receive any specific funding for this study.

## Ethics Statement

The authors have nothing to report.

## Conflicts of Interest

The authors declare no conflicts of interest.

## Supporting information


**Table S1:** Poisson regression analysis of annual trends in age‐stratified rates of overall rectal prolapse surgery in males, females, and both sexes (per 100 000 person‐years). RR, relative risk; CI, confidence interval. RR reflects the risk ratio per one‐year increase (time variable entered as continuous).
**Table S2:** Poisson regression analysis of annual trends in age‐stratified rates of conventional surgery in males, females, and both sexes (per 100 000 person‐years). RR, relative risk; CI, confidence interval. RR reflects the risk ratio per one‐year increase (time variable entered as continuous).
**Table S3:** Poisson regression analysis of annual trends in age‐stratified rates of laparoscopic surgery in males, females, and both sexes (per 100 000 person‐years). RR, relative risk; CI, confidence interval; NA, not available. RR reflects the risk ratio per one‐year increase (time variable entered as continuous).

**Figure S1:** Age‐stratified distribution of surgeries among males and females, expressed per 100 000 person‐years from 2014 to 2023: (A) conventional surgeries, (B) laparoscopic surgeries, (C) perineal surgeries, and (D) abdominal surgeries.
**Figure S2:** Relative risks (RRs) for age‐stratified conventional surgeries per 100 000 person‐years, derived from Poisson regression models: males (A), females (B) and both sexes combined (C). RR, relative risk; CI, confidence interval. Black dots represent significant, and gray dots represent non‐significant. A dashed line at RR = 1.0 represents no annual change in procedure rate.
**Figure S3:** Relative risks (RRs) for age‐stratified laparoscopic surgeries per 100 000 person‐years, derived from Poisson regression models: males (A), females (B) and both sexes combined (C). RR, relative risk; CI, confidence interval. Black dots represent significant, and gray dots represent.

## Data Availability

The data that support the findings of this study are openly available in NDB Open Data at https://www.mhlw.go.jp/stf/seisakunitsuite/bunya/0000177182.html.

## References

[1] L. Bordeianou , I. Paquette , E. Johnson , et al., “Clinical Practice Guidelines for the Treatment of Rectal Prolapse,” Diseases of the Colon and Rectum 60, no. 11 (2017): 1121–1131, 10.1097/DCR.0000000000000889. 28991074

[2] E. M. van der Schans , T. J. C. Paulides , N. A. Wijffels , and E. C. J. Consten , “Management of Patients With Rectal Prolapse: The 2017 Dutch Guidelines,” Techniques in Coloproctology 22, no. 8 (2018): 589–596, 10.1007/s10151-018-1830-1. 30099626

[3] G. Gallo , J. Martellucci , G. Pellino , et al., “Consensus Statement of the Italian Society of Colorectal Surgery (SICCR): Management and Treatment of Complete Rectal Prolapse,” Techniques in Coloproctology 22, no. 12 (2018): 919–931, 10.1007/s10151-018-1908-9. 30554284

[4] M. V. Kairaluoma and I. H. Kellokumpu , “Epidemiologic Aspects of Complete Rectal Prolapse,” Scandinavian Journal of Surgery 94, no. 3 (2005): 207–210, 10.1177/145749690509400306. 16259169

[5] Y. El‐Dhuwaib , A. Pandyan , and C. H. Knowles , “Epidemiological Trends in Surgery for Rectal Prolapse in England 2001‐2012: An Adult Hospital Population‐Based Study,” Colorectal Disease 22, no. 10 (2020): 1359–1366, 10.1111/codi.15094. 32346972

[6] E. Gialamas , I. Uhe , P. A. Tokoto , et al., “Surgeon Preferences and Practice Patterns in Rectopexy: Results of an International Survey,” Colorectal Disease 28, no. 1 (2026): e70355, 10.1111/codi.70355. 41486360 PMC12765771

[7] J. K. Kelley , E. R. Hagen , B. Gurland , A. R. Stevenson , and J. W. Ogilvie, Jr. , “The International Variability of Surgery for Rectal Prolapse,” BMJ Surgery, Interventional & Therapeutic Health Technologies 5, no. 1 (2023): e000198, 10.1136/bmjsit-2023-000198. PMC10649678 38020494

[8] Ministry of Health, Labour and Welfare , “Use of National Database of Health Insurance Claims of Japan (NDB),” (2026), https://www.mhlw.go.jp/stf/seisakunitsuite/bunya/kenkou_iryou/iryouhoken/reseputo/index.html.

[9] M. Kido , T. Arita , K. Shoda , et al., “Inter‐Prefectural and Urban‐Rural Regional Disparities in Rectal Cancer and Rectal Resections: A Japanese Nationwide Population‐Based Cohort Study From 2014 to 2019,” Annals of Gastroenterological Surgery 9, no. 2 (2024): 281–287, 10.1002/ags3.12865. 40046527 PMC11877343

[10] M. Kido , T. Arita , K. Shoda , et al., “Indirect Assessment of Hemorrhoid Incidence Using Invasive Treatment Data in Japan: A 5‐Year Study Based on Nationwide Health Insurance Claims,” Annals of Gastroenterological Surgery 9, no. 5 (2025): 987–996, 10.1002/ags3.70018. 40922913 PMC12414590

[11] M. Kido , K. Shoda , L. Yan , et al., “Inter‐ Prefectural Regional Disparities in Gastric Cancer Surgery: A Japanese Nationwide Population‐ Based Cohort Study From 2014 to 2019,” Annals of Gastroenterological Surgery 8, no. 6 (2024): 1–9, 10.1002/ags3.12813. PMC11533019 39502726

[12] M. Kido , S. Okada , N. Takashima , et al., “Inter‐Prefectural and Urban‐Rural Regional Disparities in Lung Cancer Surgery: A Japanese Nationwide Population‐Based Cohort Study From 2017 to 2019,” Surgery Today 54, no. 12 (2024): 1428–1436, 10.1007/s00595-024-02864-4. 38739174

[13] M. Kido , K. Inoue , R. Kobayashi , et al., “Seasonal Variations and a Demographic Analysis of Digital Evacuation Incidence for Constipation Management: A Japanese Population‐Based Cohort Study,” Internal Medicine 64, no. 11 (2025): 1623–1632, 10.2169/internalmedicine.4398-24. 39496447 PMC12222950

[14] Ministry of Health, Labour and Welfare , “NDB Open Data,” (2026), https://www.mhlw.go.jp/stf/seisakunitsuite/bunya/0000177182.html.

[15] “Portal Site of Official Statistics of Japan (e‐Stat),” (2026), https://www.e‐stat.go.jp/en/stat‐search/.

[16] “Japan Meteorological Agency (JMA),” (2026), https://www.jma.go.jp/jma/indexe.html.

[17] Japan Meteorological Agency (JMA) , “Past Weather Data,” (2026), https://www.data.jma.go.jp/gmd/risk/obsdl/index.php.

[18] Y. Kanda , “Investigation of the Freely Available Easy‐To‐Use Software ‘EZR’ for Medical Statistics,” Bone Marrow Transplantation 48, no. 3 (2013): 452–458, 10.1038/bmt.2012.244. 23208313 PMC3590441

[19] J. O. L. DeLancey , M. Masteling , F. Pipitone , J. LaCross , S. Mastrovito , and J. A. Ashton‐Miller , “Pelvic Floor Injury During Vaginal Birth Is Life‐Altering and Preventable: What Can We Do About It?,” American Journal of Obstetrics and Gynecology 230, no. 3 (2024): 279–294, 10.1016/j.ajog.2023.11.1253. 38168908 PMC11177602

[20] R. Oizumi , H. Inaba , T. Takada , Y. Enatsu , and K. Kinjo , “Sensitivity Analysis on the Declining Population in Japan: Effects of Prefecture‐Specific Fertility and Interregional Migration,” PLoS One 17, no. 9 (2022): e0273817, 10.1371/journal.pone.0273817. 36103457 PMC9473415

[21] K. Ishikawa , T. Ikeda , and R. Miyazaki , “Cesarean Delivery and Perinatal Mortality Rates in Japan, 2007‐2011,” Journal of Maternal‐Fetal & Neonatal Medicine 27, no. 10 (2014): 994–999, 10.3109/14767058.2013.847422. 24060236

[22] T. Boerma , C. Ronsmans , D. Y. Melesse , et al., “Global Epidemiology of Use of and Disparities in Caesarean Sections,” Lancet 392, no. 10155 (2018): 1341–1348, 10.1016/S0140-6736(18)31928-7. 30322584

[23] M. Alperin and A. Artsen , “Cesarean Delivery on Maternal Request,” Journal of the American Medical Association 335, no. 10 (2026): 903–904, 10.1001/jama.2025.26633. 41661634 PMC13316488

[24] T. Bjerke and T. Mynster , “One Decade of Rectal Prolapse Surgery: A National Study,” International Journal of Colorectal Disease 33, no. 3 (2018): 299–304, 10.1007/s00384-017-2944-z. 29273884

[25] A. C. Rogers , N. McCawley , A. M. Hanly , J. Deasy , D. A. McNamara , and J. P. Burke , “Trends in the Treatment of Rectal Prolapse: A Population Analysis,” International Journal of Colorectal Disease 33, no. 4 (2018): 459–465, 10.1007/s00384-018-2971-4. 29502314

[26] V. T. Daniel , J. S. Davids , P. R. Sturrock , J. A. Maykel , U. R. Phatak , and K. Alavi , “Getting to the Bottom of Treatment of Rectal Prolapse in the Elderly: Analysis of the National Surgical Quality Improvement Program (NSQIP),” American Journal of Surgery 218, no. 2 (2019): 288–292, 10.1016/j.amjsurg.2019.02.010. 30803700

[27] A. Hotouras , J. Murphy , A. Abeles , et al., “Symptom Distribution and Anorectal Physiology Results in Male Patients With Rectal Intussusception and Prolapse,” Journal of Surgical Research 188, no. 1 (2014): 298–302, 10.1016/j.jss.2013.12.008. 24411299

