# Supplementary material for: Trends, Demographic Characteristics and Seasonal Patterns of Rectal Prolapse Surgery in Japan: A Nationwide Claims‐Based Analysis From 2014 to 2023
Source: Ann Gastroenterol Surg. 2026 Jun 26;10(5):1612–22. doi: 10.1002/ags3.70242 (PMC13394726; doi:10.1002/ags3.70242)

A **Supple Figure 1**

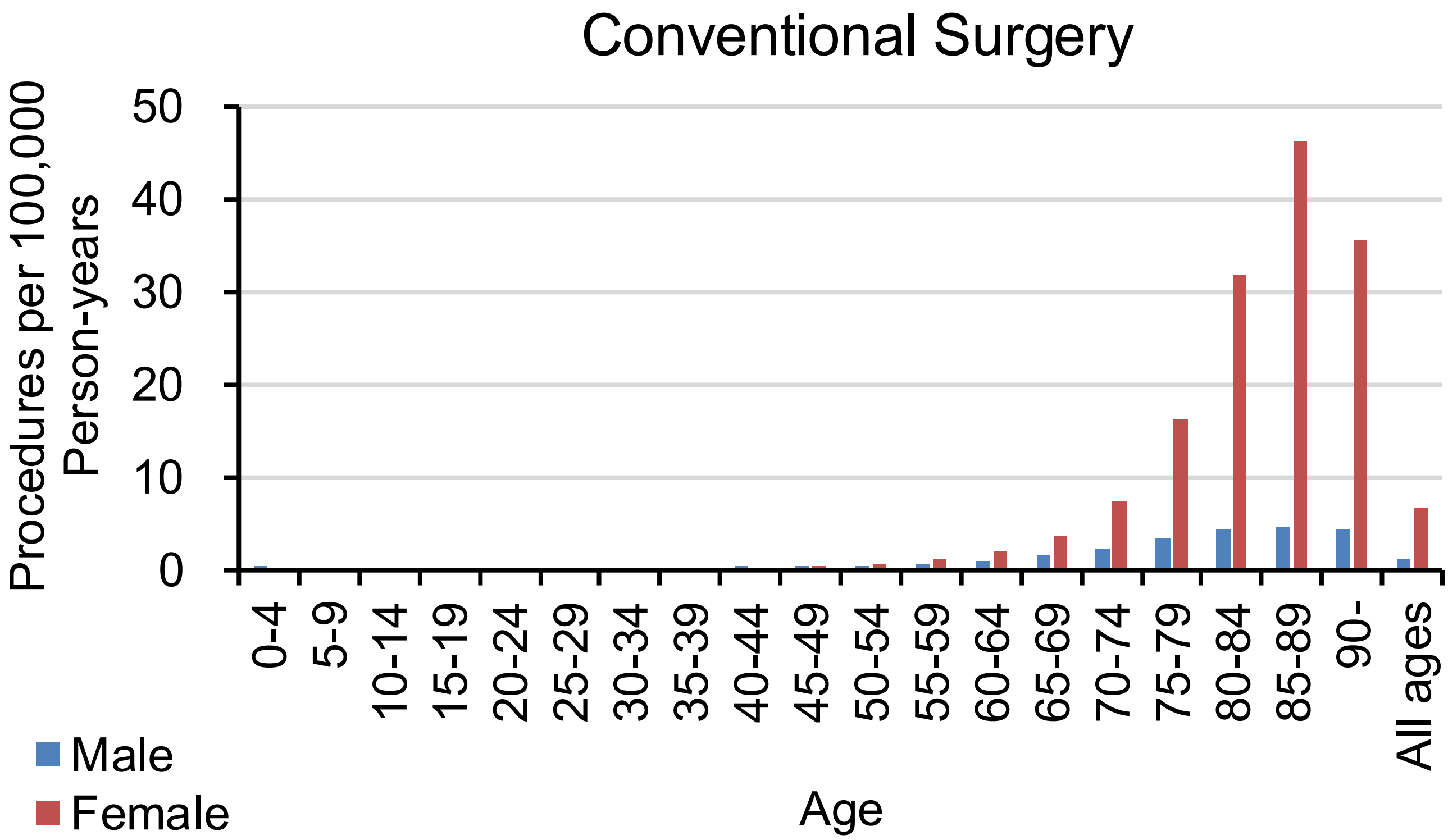

B

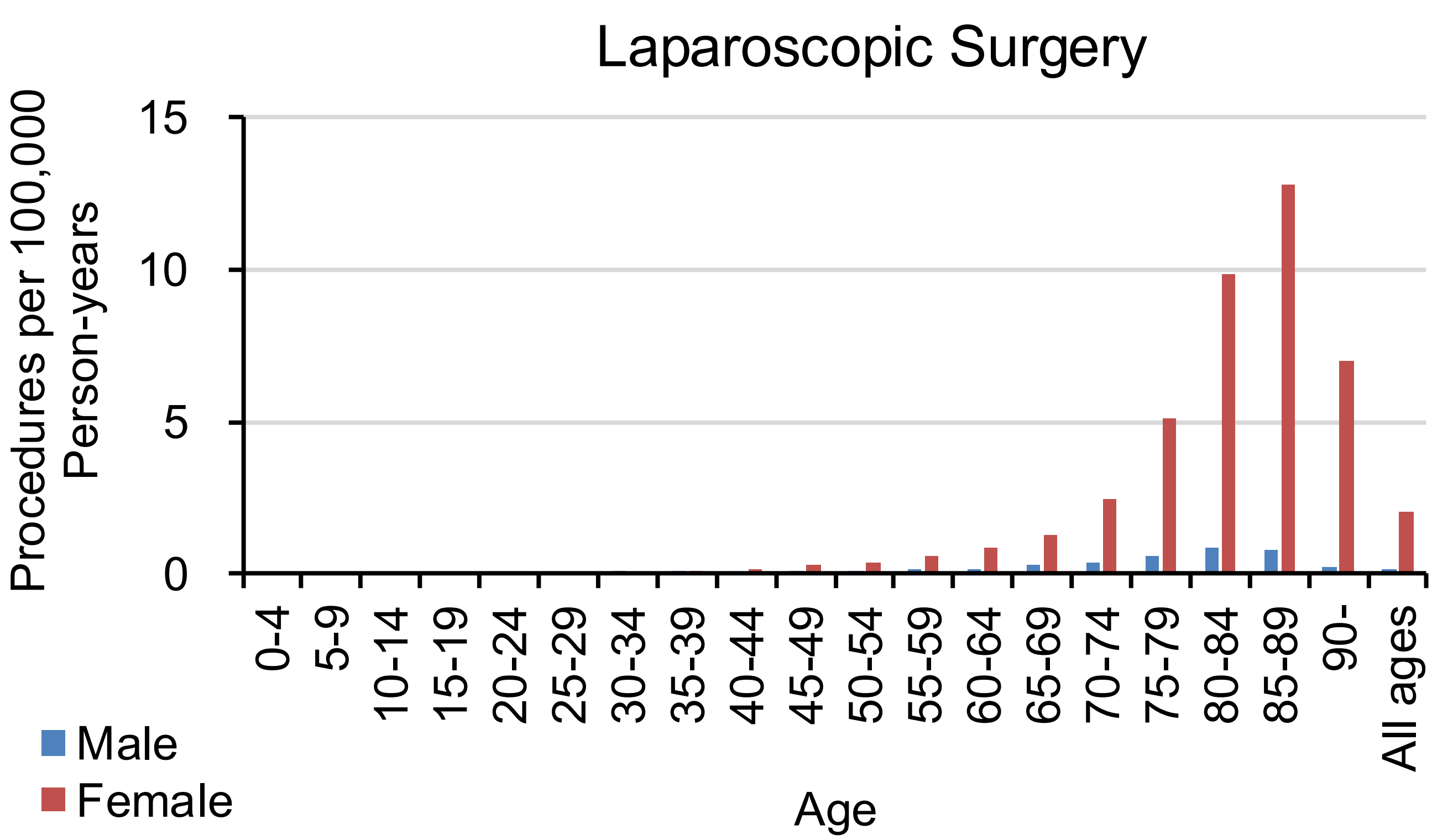

C **Supple Figure 1**

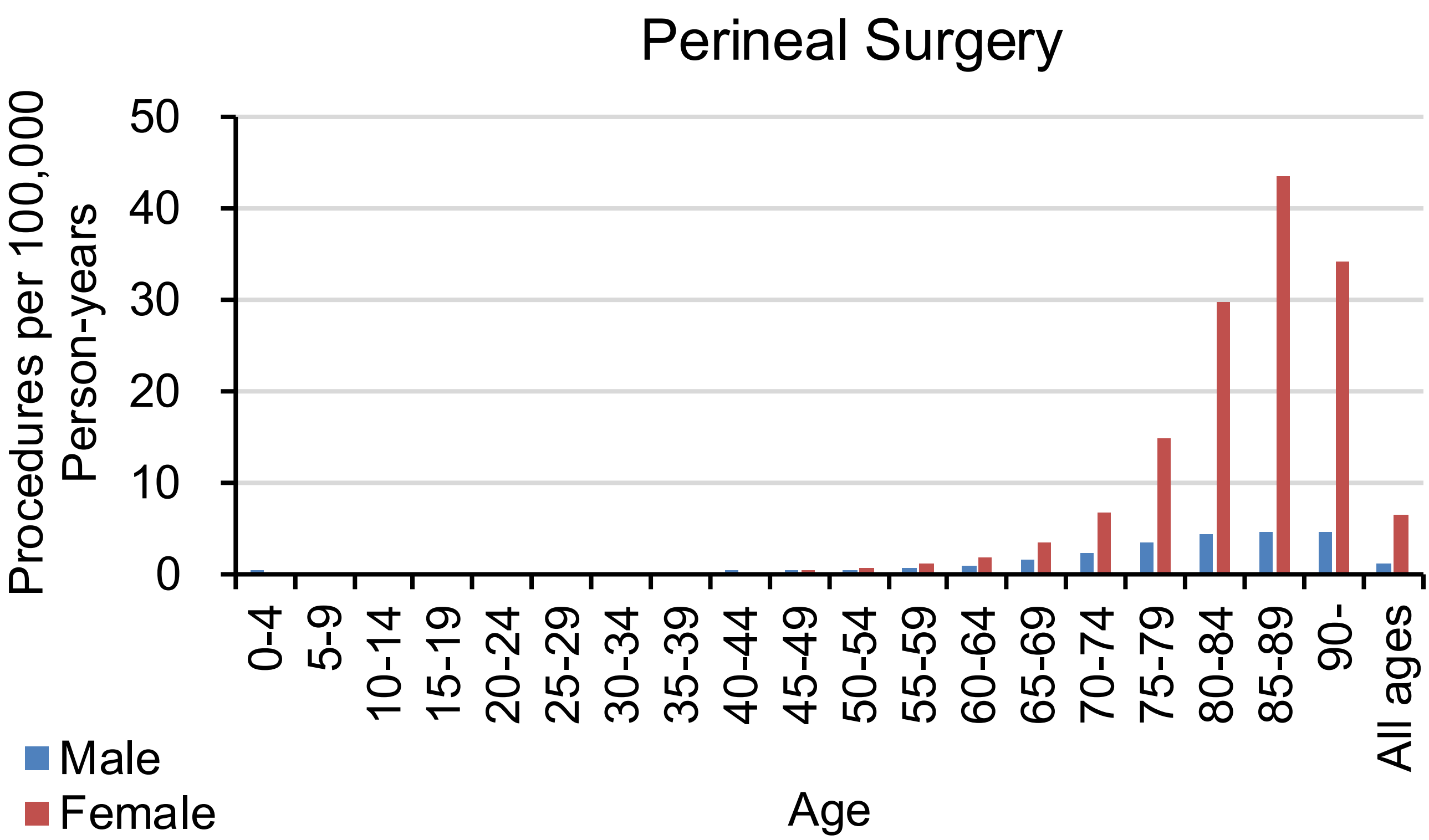

D

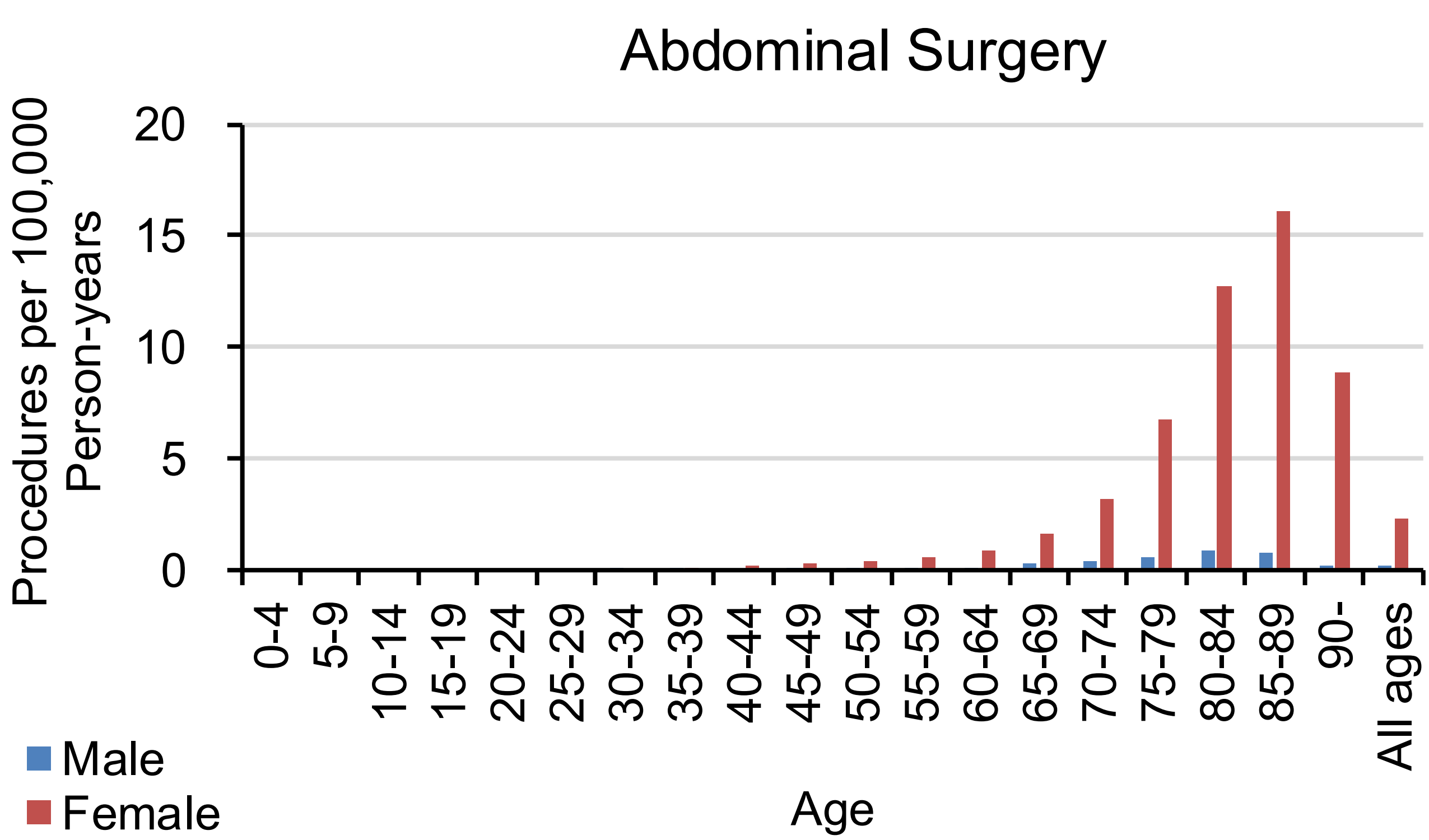

Supple Figure 2

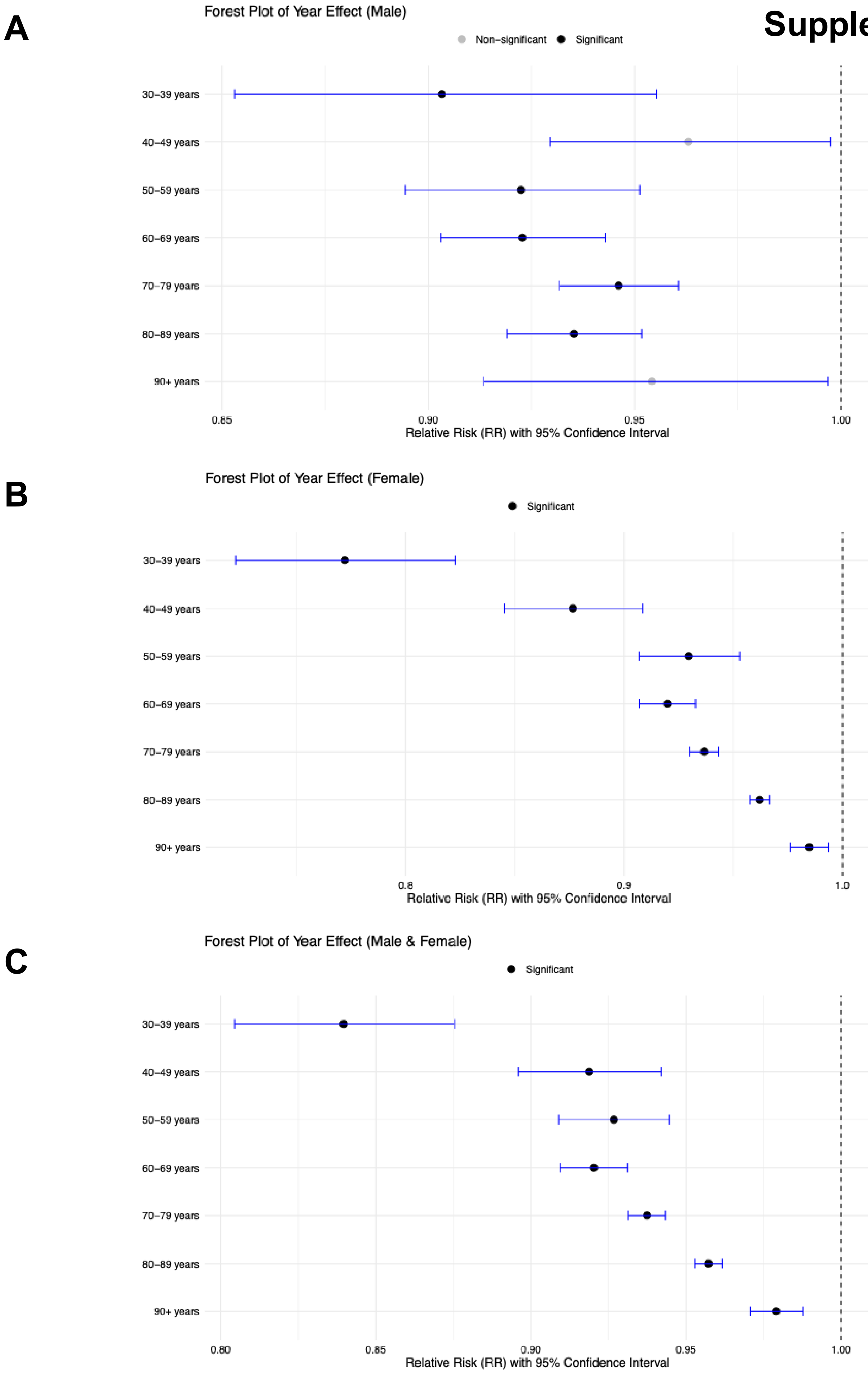

Supple Figure 3

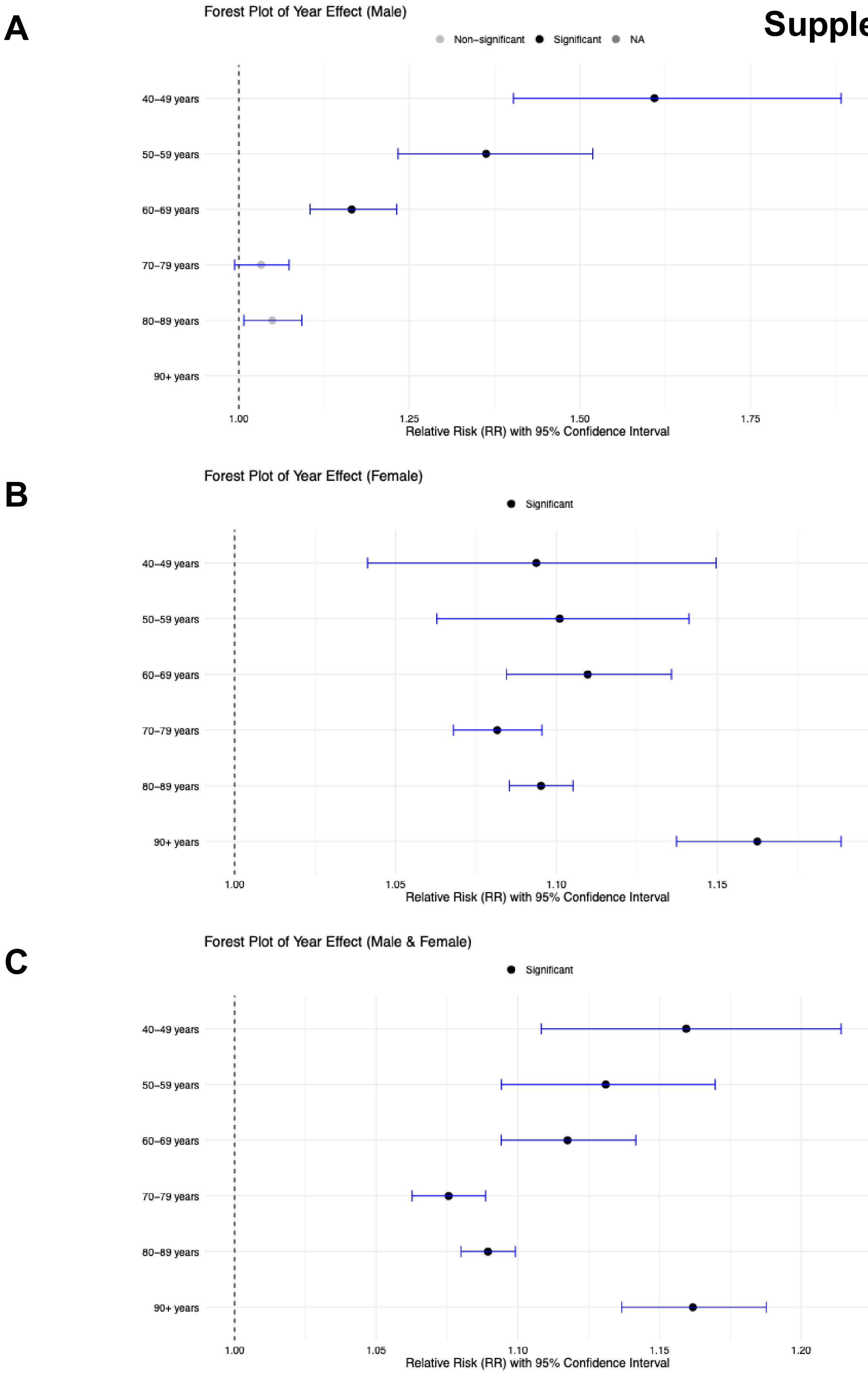

Supplement: Supplementary file 1 [file AGS3-10-1612-s001.pdf]
