# Supplementary material for: Trends, Demographic Characteristics and Seasonal Patterns of Rectal Prolapse Surgery in Japan: A Nationwide Claims‐Based Analysis From 2014 to 2023
Source: Ann Gastroenterol Surg. 2026 Jun 26:10.1002/ags3.70242. Online ahead of print. doi: 10.1002/ags3.70242 (PMC13394726; doi:10.1002/ags3.70242)
Supplement: Supplementary file 1 — Table S1: Poisson regression analysis of annual trends in age‐stratified rates of overall rectal prolapse surgery in males, females, and both sexes (per 100 000 person‐years). RR, relative risk; CI, confidence interval. RR reflects the risk ratio per one‐year increase (time variable entered as continuous). Table S2: Poisson regression analysis of annual trends in age‐stratified rates of conventional surgery in males, females, and both sexes (per 100 000 person‐years). RR, relative risk; CI, confidence interval. RR reflects the risk ratio per one‐year increase (time variable entered as continuous). Table S3: Poisson regression analysis of annual trends in age‐stratified rates of laparoscopic surgery in males, females, and both sexes (per 100 000 person‐years). RR, relative risk; CI, confidence interval; NA, not available. RR reflects the risk ratio per one‐year increase (time variable entered as continuous). [file AGS3-9999-0-s002.docx]

**Supplementary Table 1.** Poisson regression analysis of annual trends in age-stratified rates of overall rectal prolapse surgery in males, females, and both sexes (per 100,000 person-years).

| **Sex** | **Age Group** | **RR** | **95% CI (low)** | **95% CI (high)** | ***P*-value** |
| --- | --- | --- | --- | --- | --- |
| Male | 30-39 years | 0.996 | 0.947 | 1.046 | 0.8598 |
| Male | 40-49 years | 1.009 | 0.976 | 1.042 | 0.6113 |
| Male | 50-59 years | 0.962 | 0.935 | 0.990 | 0.0082 |
| Male | 60-69 years | 0.954 | 0.935 | 0.973 | < 0.0001 |
| Male | 70-79 years | 0.957 | 0.944 | 0.971 | < 0.0001 |
| Male | 80-89 years | 0.952 | 0.937 | 0.968 | < 0.0001 |
| Male | 90+ years | 0.978 | 0.937 | 1.021 | 0.3061 |
| Female | 30-39 years | 0.757 | 0.709 | 0.806 | < 0.0001 |
| Female | 40-49 years | 0.946 | 0.919 | 0.973 | 0.0001 |
| Female | 50-59 years | 0.984 | 0.964 | 1.004 | 0.1062 |
| Female | 60-69 years | 0.967 | 0.956 | 0.979 | < 0.0001 |
| Female | 70-79 years | 0.970 | 0.964 | 0.975 | < 0.0001 |
| Female | 80-89 years | 0.990 | 0.986 | 0.994 | < 0.0001 |
| Female | 90+ years | 1.010 | 1.002 | 1.019 | 0.0141 |
| Male&Female | 30-39 years | 0.886 | 0.853 | 0.920 | < 0.0001 |
| Male&Female | 40-49 years | 0.972 | 0.951 | 0.993 | 0.0087 |
| Male&Female | 50-59 years | 0.976 | 0.960 | 0.992 | 0.0041 |
| Male&Female | 60-69 years | 0.963 | 0.953 | 0.973 | < 0.0001 |
| Male&Female | 70-79 years | 0.967 | 0.961 | 0.972 | < 0.0001 |
| Male&Female | 80-89 years | 0.985 | 0.981 | 0.989 | < 0.0001 |
| Male&Female | 90+ years | 1.005 | 0.996 | 1.013 | 0.2709 |

Note: RR, relative risk; CI, confidence interval. RR reflects the risk ratio per one-year increase (time variable entered as continuous).

**Supplementary Table 2.** Poisson regression analysis of annual trends in age-stratified rates of conventional surgery in males, females, and both sexes (per 100,000 person-years).

| **Sex** | **Age Group** | **RR** | **95% CI (low)** | **95% CI (high)** | ***P*-value** |
| --- | --- | --- | --- | --- | --- |
| Male | 30-39 years | 0.903 | 0.853 | 0.955 | 0.0004 |
| Male | 40-49 years | 0.963 | 0.930 | 0.997 | 0.0357 |
| Male | 50-59 years | 0.923 | 0.894 | 0.951 | < 0.0001 |
| Male | 60-69 years | 0.923 | 0.903 | 0.943 | < 0.0001 |
| Male | 70-79 years | 0.946 | 0.932 | 0.961 | < 0.0001 |
| Male | 80-89 years | 0.935 | 0.919 | 0.952 | < 0.0001 |
| Male | 90+ years | 0.954 | 0.913 | 0.997 | 0.0351 |
| Female | 30-39 years | 0.772 | 0.722 | 0.823 | < 0.0001 |
| Female | 40-49 years | 0.877 | 0.845 | 0.909 | < 0.0001 |
| Female | 50-59 years | 0.930 | 0.907 | 0.953 | < 0.0001 |
| Female | 60-69 years | 0.920 | 0.907 | 0.933 | < 0.0001 |
| Female | 70-79 years | 0.937 | 0.930 | 0.943 | < 0.0001 |
| Female | 80-89 years | 0.962 | 0.958 | 0.967 | < 0.0001 |
| Female | 90+ years | 0.985 | 0.976 | 0.994 | 0.0008 |
| Male&Female | 30-39 years | 0.840 | 0.804 | 0.875 | < 0.0001 |
| Male&Female | 40-49 years | 0.919 | 0.896 | 0.942 | < 0.0001 |
| Male&Female | 50-59 years | 0.927 | 0.909 | 0.945 | < 0.0001 |
| Male&Female | 60-69 years | 0.920 | 0.910 | 0.931 | < 0.0001 |
| Male&Female | 70-79 years | 0.937 | 0.931 | 0.943 | < 0.0001 |
| Male&Female | 80-89 years | 0.957 | 0.953 | 0.962 | < 0.0001 |
| Male&Female | 90+ years | 0.979 | 0.971 | 0.988 | < 0.0001 |

Note: RR, relative risk; CI, confidence interval. RR reflects the risk ratio per one-year increase (time variable entered as continuous).

**Supplementary Table 3.** Poisson regression analysis of annual trends in age-stratified rates of laparoscopic surgery in males, females, and both sexes (per 100,000 person-years).

| **Sex** | **Age Group** | **RR** | **95% CI (low)** | **95% CI (high)** | ***P*-value** |
| --- | --- | --- | --- | --- | --- |
| Male | 40-49 years | 1.609 | 1.402 | 1.883 | < 0.0001 |
| Male | 50-59 years | 1.362 | 1.233 | 1.519 | < 0.0001 |
| Male | 60-69 years | 1.166 | 1.105 | 1.231 | < 0.0001 |
| Male | 70-79 years | 1.033 | 0.994 | 1.074 | 0.0994 |
| Male | 80-89 years | 1.049 | 1.008 | 1.093 | 0.0207 |
| Male | 90+ years | NA | NA | NA | NA |
| Female | 40-49 years | 1.094 | 1.041 | 1.150 | 0.0004 |
| Female | 50-59 years | 1.101 | 1.063 | 1.141 | < 0.0001 |
| Female | 60-69 years | 1.110 | 1.084 | 1.136 | < 0.0001 |
| Female | 70-79 years | 1.082 | 1.068 | 1.095 | < 0.0001 |
| Female | 80-89 years | 1.095 | 1.085 | 1.105 | < 0.0001 |
| Female | 90+ years | 1.162 | 1.137 | 1.188 | < 0.0001 |
| Male&Female | 40-49 years | 1.159 | 1.108 | 1.214 | < 0.0001 |
| Male&Female | 50-59 years | 1.131 | 1.094 | 1.170 | < 0.0001 |
| Male&Female | 60-69 years | 1.118 | 1.094 | 1.142 | < 0.0001 |
| Male&Female | 70-79 years | 1.076 | 1.063 | 1.089 | < 0.0001 |
| Male&Female | 80-89 years | 1.089 | 1.080 | 1.099 | < 0.0001 |
| Male&Female | 90+ years | 1.162 | 1.137 | 1.188 | < 0.0001 |

Note: RR, relative risk; CI, confidence interval; NA, not available. RR reflects the risk ratio per one-year increase (time variable entered as continuous).
