# Supplementary figures and images for: Trends, Demographic Characteristics and Seasonal Patterns of Rectal Prolapse Surgery in Japan: A Nationwide Claims‐Based Analysis From 2014 to 2023
Source: Ann Gastroenterol Surg. 2026 Jun 26:10.1002/ags3.70242. Online ahead of print. doi: 10.1002/ags3.70242 (PMC13394726; doi:10.1002/ags3.70242)

**A****Conventional Surgery**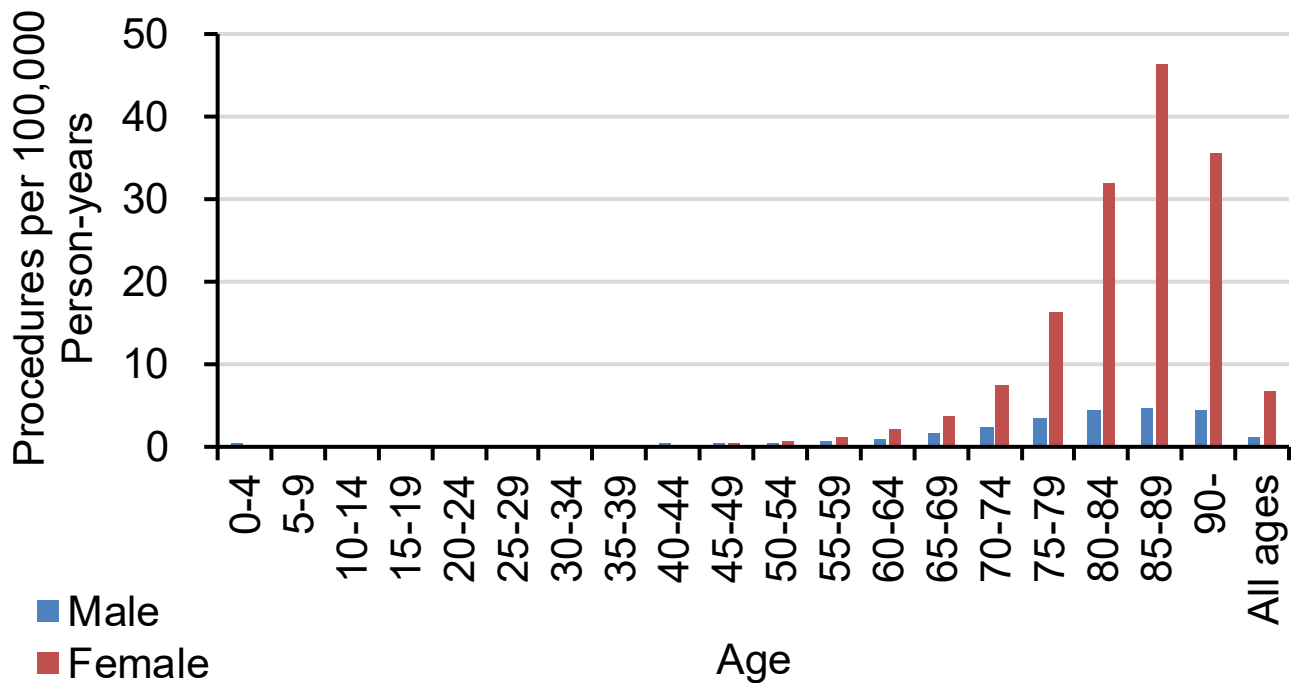**B****Laparoscopic Surgery**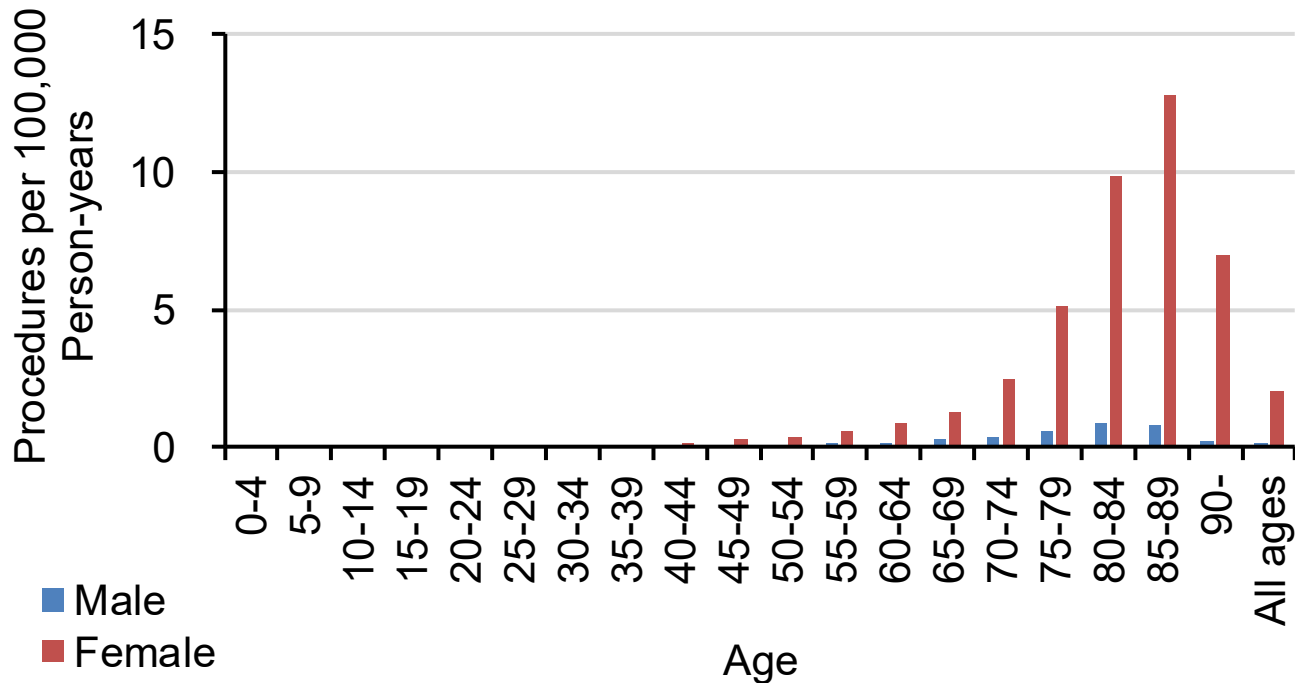

C

Perineal Surgery

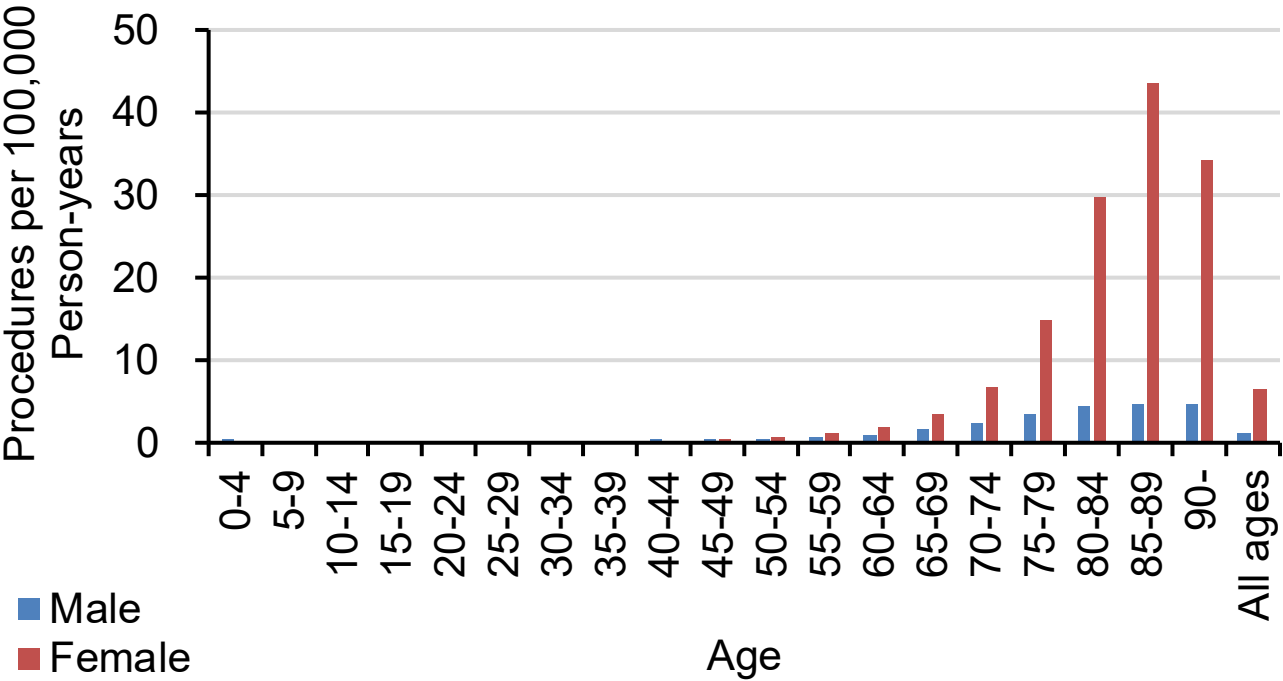

D

Abdominal Surgery

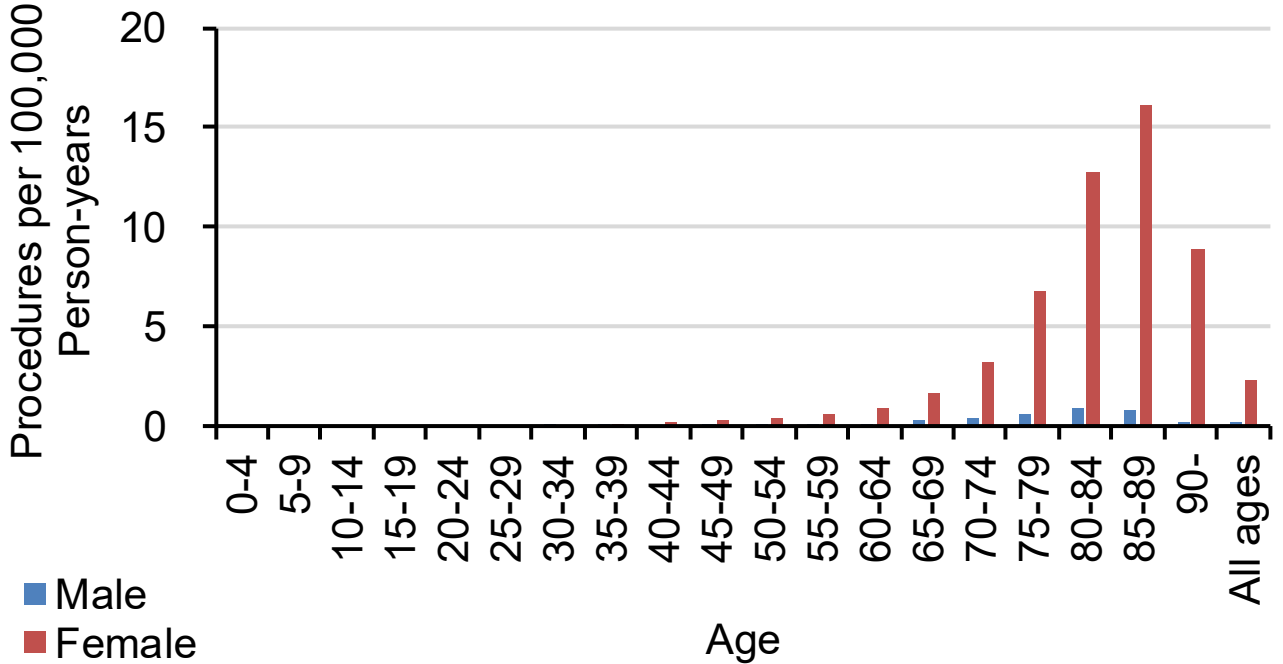

## A

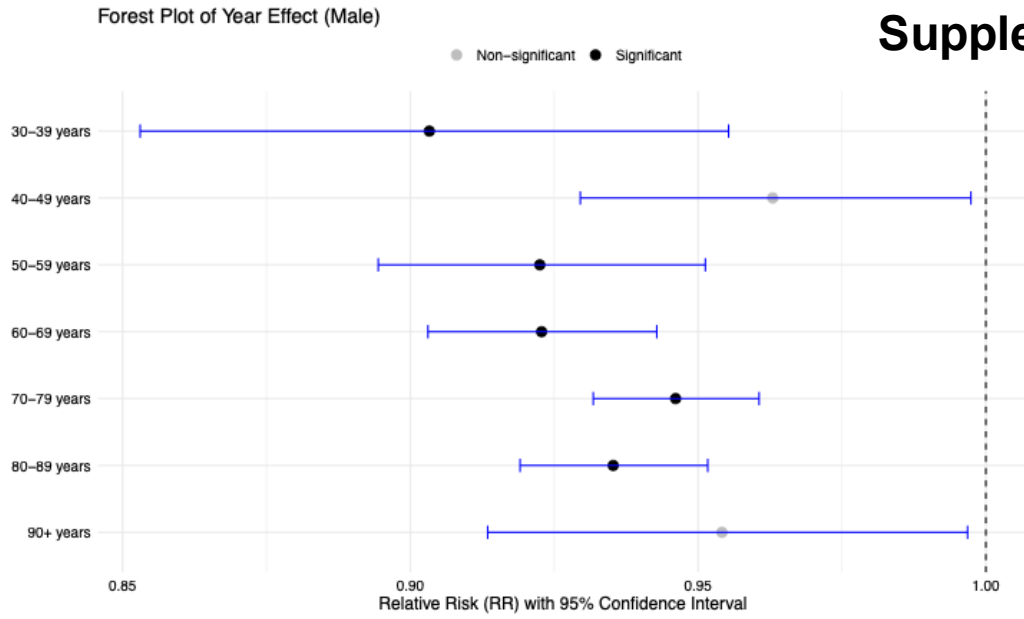

## B

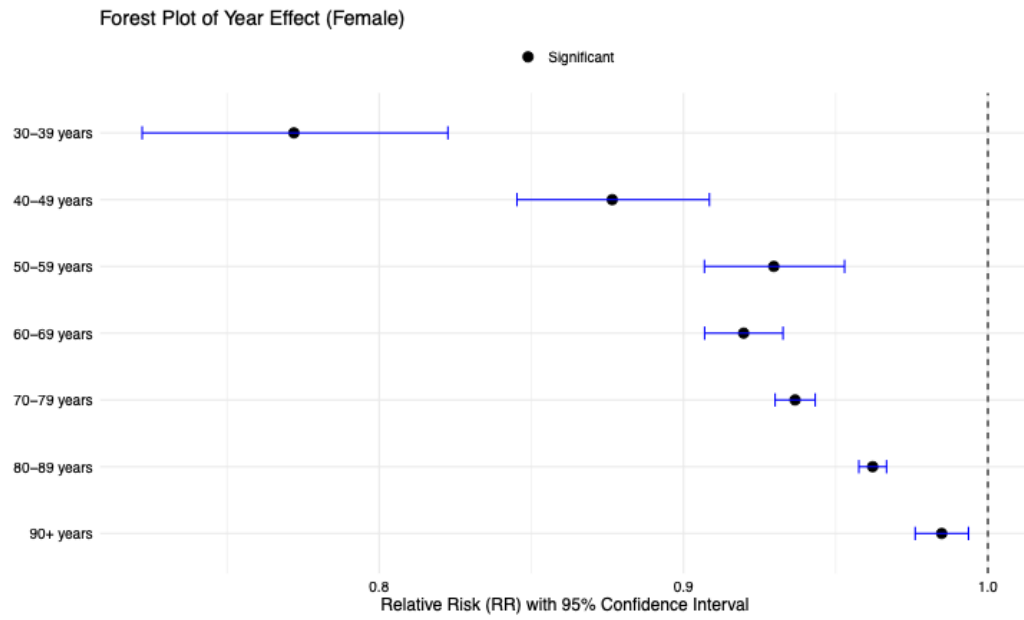

## C

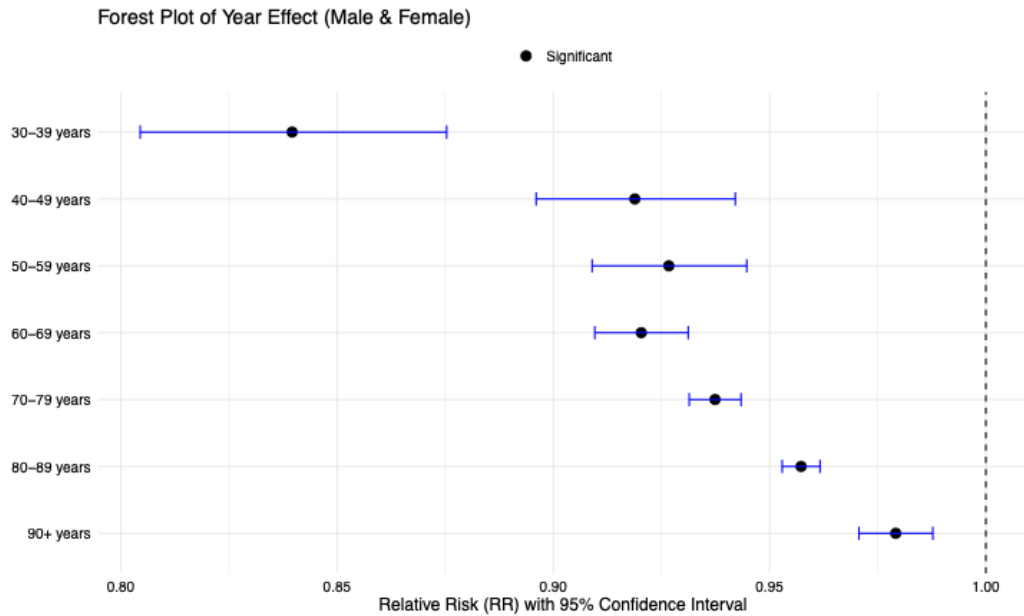

A

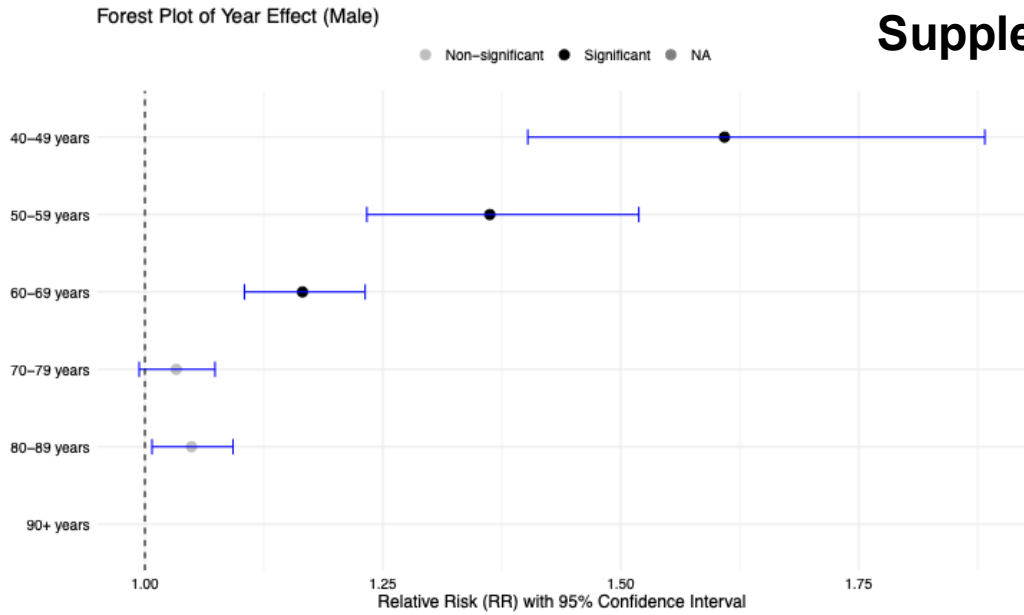

B

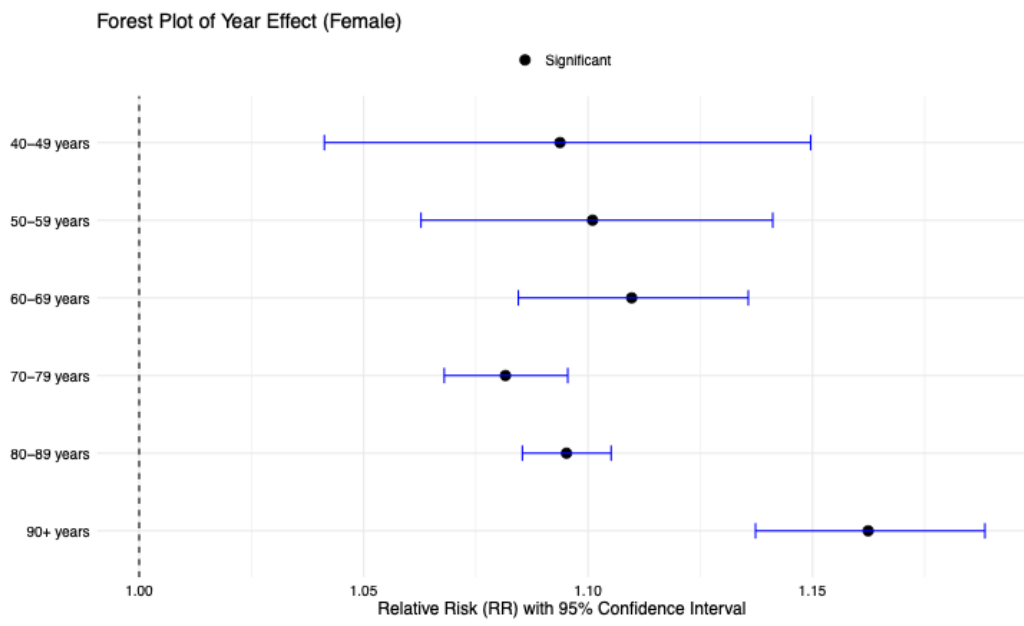

C

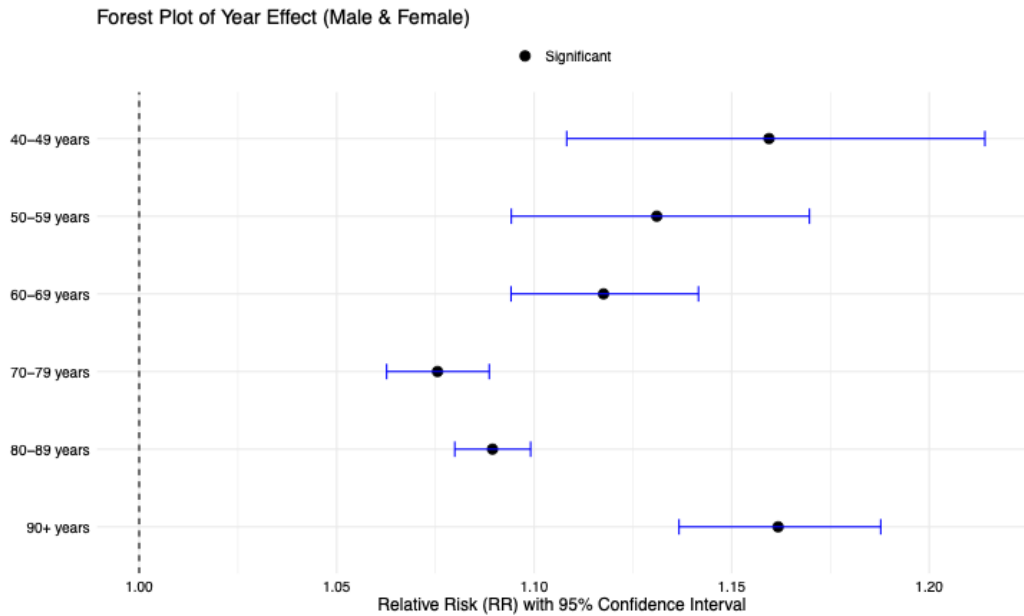

Supplement: Supplementary file 2 — Figure S1: Age‐stratified distribution of surgeries among males and females, expressed per 100 000 person‐years from 2014 to 2023: (A) conventional surgeries, (B) laparoscopic surgeries, (C) perineal surgeries, and (D) abdominal surgeries. Figure S2: Relative risks (RRs) for age‐stratified conventional surgeries per 100 000 person‐years, derived from Poisson regression models: males (A), females (B) and both sexes combined (C). RR, relative risk; CI, confidence interval. Black dots represent significant, and gray dots represent non‐significant. A dashed line at RR = 1.0 represents no annual change in procedure rate. Figure S3: Relative risks (RRs) for age‐stratified laparoscopic surgeries per 100 000 person‐years, derived from Poisson regression models: males (A), females (B) and both sexes combined (C). RR, relative risk; CI, confidence interval. Black dots represent significant, and gray dots represent. [file AGS3-9999-0-s001.pdf]
